# Molecular recognition of *N*-acetyltryptophan enantiomers by β-cyclodextrin

**DOI:** 10.3762/bjoc.13.157

**Published:** 2017-08-09

**Authors:** Spyros D Chatziefthimiou, Mario Inclán, Petros Giastas, Athanasios Papakyriakou, Konstantina Yannakopoulou, Irene M Mavridis

**Affiliations:** 1 Institute of Nanoscience & Nanotechnology, National Center for Scientific Research “Demokritos”, Patriarchou Gregoriou E’ & Neapoleos 27, 15310 Aghia Paraskevi Attikis, Greece

**Keywords:** β-cyclodextrin, enantiomeric discrimination, *N*-acetyltryptophan, NMR, X-ray structure

## Abstract

The enantioselectivity of β-cyclodextrin (β-CD) towards L- and D-*N*-acetyltryptophan (NAcTrp) has been studied in aqueous solution and the crystalline state. NMR studies in solution show that β-CD forms complexes of very similar but not identical geometry with both L- and D-NAcTrp and exhibits stronger binding with L-NAcTrp. In the crystalline state, only β-CD–L-NAcTrp crystallizes readily from aqueous solutions as a dimeric complex (two hosts enclosing two guest molecules). In contrast, crystals of the complex β-CD–D-NAcTrp were never obtained, although numerous conditions were tried. In aqueous solution, the orientation of the guest in both complexes is different than in the β-CD–L-NAcTrp complex in the crystal. Overall, the study shows that subtle differences observed between the β-CD–L,D-NAcTrp complexes in aqueous solution are magnified at the onset of crystallization, as a consequence of accumulation of many soft host–guest interactions and of the imposed crystallographic order, thus resulting in very dissimilar propensity of each enantiomer to produce crystals with β-CD.

## Introduction

Cyclodextrins (CDs) are cyclic, water-soluble carbohydrates with a rather non-polar cavity that can host a variety of organic molecules (guests) and form inclusion complexes [1]. The guest molecules may be completely or partly enclosed inside the cavity depending on their size and the CD macrocycle’s dimensions. The host–guest interactions established in the cavity are of van der Waals type, whereas between parts of the guest extending out of the cavity and the host’s hydroxy groups are H-bonding interactions and/or of electrostatic nature. CDs have been studied and used for the enhancement of solubility, bioavailability and stability of drugs [2–5]. Moreover, being oligomers of α-D-glucopyranose, CDs possess an intrinsic chirality, thus they form diastereomeric inclusion complexes with enantiomeric pairs and frequently they exhibit enantioselectivity in aqueous solution or they can co-precipitate with only one enantiomer (enantioseparation). The separation of enantiomers via cyclodextrin inclusion is particularly important in the case of guests of pharmaceutical interest, since enantiomerically pure drugs are crucial for the pharmaceutical industry [1,6–7].

It has been proven difficult so far to explain and to predict the recognition abilities of specific CDs towards enantiomers, especially in solution. An interesting attempt is a thermodynamic study in aqueous solution with microcalorimetry of a large number (43) and variety of chiral organic compounds [8] with β-CD at room temperature. It was shown that properties and interactions important for chiral recognition include (i) weak non-bonding interactions rather than polar, (ii) nonsymmetrical non-polar penetrating guests and (iii) large distance of the chiral center from charged/hydrophilic groups. Moreover, trends in enantioselectivity do not follow trends in association constants, i.e., the association constants for the β-CD complexes of both enantiomers of *N*-acetyltyrosine, *N*-acetylphenylalanine and *N*-acetyltryptophan are in decreasing order, whereas their enantioselectivity (ratio of the binding constants, *K*, of the L- to the D-enantiomer) shows an increasing order (1.04, 1.1 and 1.34, respectively). X-ray crystallography, on the other hand, can improve our understanding of chiral recognition by CDs at the atomic level by providing insight into the interactions and the fit of the guest in the cavity, taking into account that crystal lattice forces may introduce additional and more stringent parameters for the enantiodiscrimination [9–10]. However, the crystallographic structures of diastereomeric complexes of CDs with chiral guest molecules in the literature are scarce. For β-CD with fenoprofen [7], a partial chiral resolution of the racemic mixture occurs, since the obtained crystals contain discrete β-CD dimers enclosing (*R*)- or (*S*)-enantiomers in a *S*/*R* ratio = 3:1. The enantiomers adopt different orientations in the β-CD dimers and preference of the (*S*) complex is dictated both by stronger H-bonding of the carboxyl group, as well as more favorable methyl–phenyl interactions inside the cavity. In contrast, no discrimination is shown by β-CD for (*R*)- and (*S*)-flurbiprofen [11], since the crystals grown from the racemic mixture have both enantiomers enclosed (as a head-to-head dimer) in a β-CD dimer. In the case of substituted CDs, 2,3,6-tri-*O*-methyl-α-CD discriminates between (*R*)- and (*S*)-mandelic acid [12] as it forms very different crystals from a racemic mixture. The same host crystallizes exclusively with (*R*)-(−)-1,7-dioxaspiro[5.5]undecane, the *Dacus Oleae* pheromone, from an aqueous solution of the racemic mixture (enantioseparation) [13] also exhibiting high enantioselectivity in solution. Likewise, heptakis-(2,3,6-tri-*O*-methyl)-β-CD displays high enantioselectivity in solution towards (*S*)-(+)-1,7-dioxaspiro[5.5]undecane and under certain conditions it co-crystallizes only with the (*S*)-enantiomer [14]. Induced host–guest fit, made possible by the macrocyclic flexibility of the permethylated CDs plays a crucial role in their capacity for chiral discrimination.

**Scheme 1:**
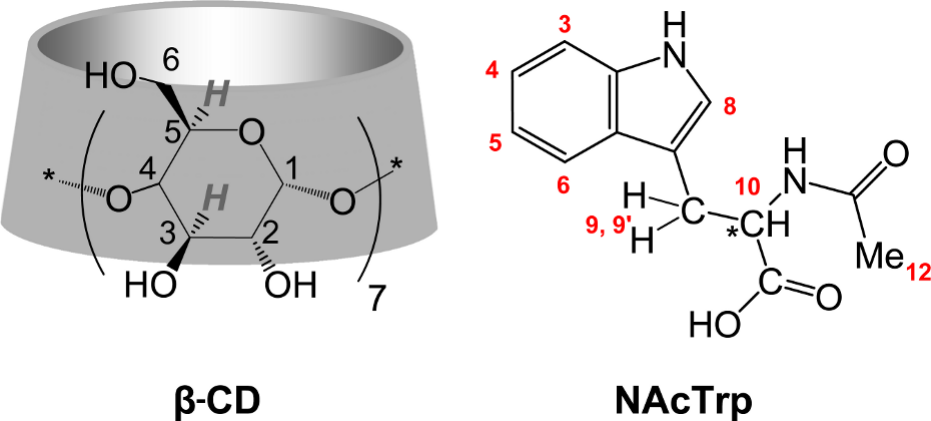
Numbering scheme of one glucopyranose residue (G) of β-CD and the NAcTrp molecule; specific atom labels of β-CD are denoted in the text/tables as Cmn, Omn, m being the atom number and n the glucopyranose residue (Gn) of β-CD.

Chiral recognition of amino acids and their derivatives by CDs has been also tested using phase-solubility diagrams [15], NMR spectroscopy [16] and electrochemical methods [17], as well as by X-ray crystallography [18]. Detailed structures of β-CD with L- and D-*N*-acetylphenylalanine (NAcPhe) grown separately [18] has shown that although the two complexes are isomorphous (same space group, very similar unit cell dimensions and same packing of β-CD dimers) there are differences regarding the positioning of the guest molecules, the D-enantiomer being ordered, whereas the L- enantiomer extensively disordered. This disparity seems to be determined by subtle hydrophobic differences and H-bonding interactions among guests themselves and with the host and co-crystallized water molecules in the lattice. Additional structures of β-CD with different L-phenylalanine derivatives [19–20] confirm the above general result. In the present study, we report on the inclusion of the L- and D-enantiomers of *N*-acetyltryptophan (NAcTrp) in β-CD (Scheme 1) in an effort to contribute to the study of chiral recognition of amino acid derivatives by CDs in the crystalline state and in solution. The guest NAcTrp has been selected because of its large aromatic side chain with appropriate dimensions to fit tightly in the β-CD cavity thus expected to have restricted mobility and limited disorder. Indicative of the interest and possible applications of the CD use in chiral selectivity/discrimination of tryptophan are studies in aqueous solution [21], in electrochemistry for sensor development [17,22–23], as components of solid phases in chromatography [24], or in capillary electrophoresis [25].

## Results and Discussion

### NMR studies

In deuterium oxide (D_2_O), each of the NAcTrp enantiomers induced significant chemical shift displacements (shielding) in the ^1^H NMR signals of the β-CD cavity protons, namely H3 (near the wider, secondary side) and H5, H6,6’ (at the narrower, primary side), signifying cavity inclusion of each enantiomer (Scheme 1). When a racemic mixture of NAcTrp was added to a β-CD solution no differentiation in the signals was observed due to in situ formation of diastereomers, except for a very small splitting of the methyl signal of the *N*-acetyl group. No differentiation was detected in the ^13^C NMR spectrum either. In order to determine the stoichiometry of the complexes continuous variation (Job) plots [26] were drafted. For β-CD protons only the cavity signals due to H5, H3 and H6,6’ showed significant shifts upon complexation (Supporting Information File 1, Figure S1). The inflection point of the graphs at 0.5 indicates a 1:1 stoichiometry for both enantiomers. The tryptophan protons were affected differently upon complexation (Supporting Information File 1, Figure S2), i.e., the graphs due to shifts of the indole’s benzene ring protons (H3, H4, H5 and H6) indicate a 1:1 host/guest stoichiometry, whereas those of the indole moiety (H8) and of the aliphatic protons (H9,9’, H10, H12), with an inflection point at ≈0.3, suggest a host/guest ratio close to 2:1. This behavior reveals the existence of two different complexation modes, one involving the indole phenyl ring with one host only and the aliphatic chain with two host molecules. The fact that the second mode takes place mainly when there is an excess of host concentration indicates that the inclusion of the indole moiety is the predominant mode of interaction. Moreover, it is observed that the magnitude of the shifts of the L-enantiomer are always larger and the slopes of the Job plots steeper than those of the D-enantiomer, suggesting stronger binding of β-CD with L- than with D-NAcTrp.

**Figure 1:**
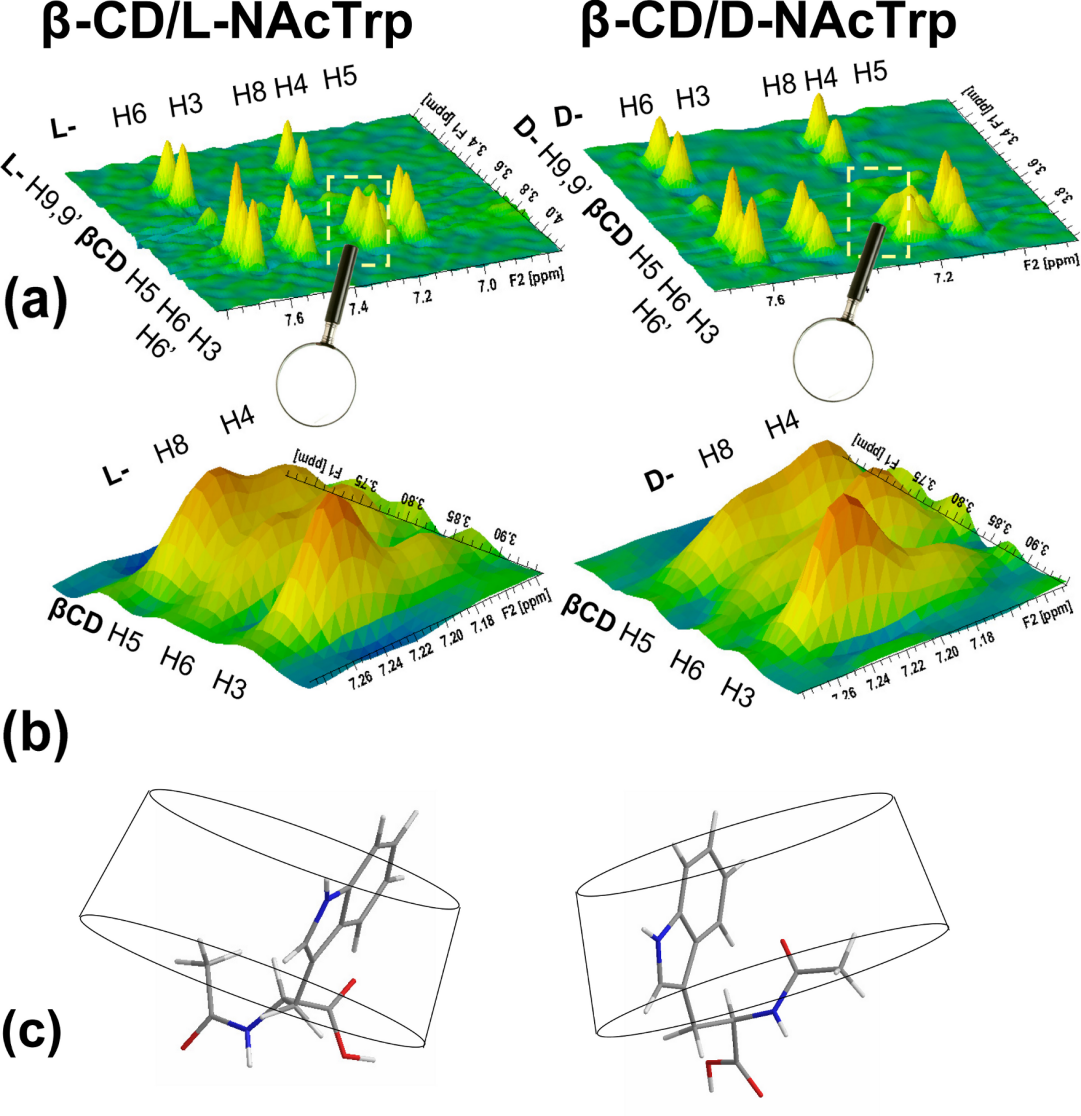
3D maps of the observed dipolar, through-space host–guest interactions depicted so as to (a) reflect ≈equally strong cross-peaks in each L- and D-NAcTrp enantiomer for intramolecular correlations, namely guest H9,9’ with guest H6 and H8; (b) the few differences observed in the intermolecular interactions have been magnified and reflect somewhat different binding modes of the two enantiomers revealed by intermolecular interactions of guest protons H8 and H4 only; (c) schematic representation of the respective solution models for each 1:1 complex.

2D ROESY spectra of each enantiomer with β-CD at a 1:1 mole ratio in D_2_O were obtained under identical conditions (temperature, concentration, acquisition parameters). Strong intermolecular dipolar interactions were observed between indole protons (H3, H4, H5, H6, H8) and the β-CD cavity protons (H5, H6,6’, H3) in both enantiomeric guests, confirming full inclusion of the Trp side chain. To facilitate the comparison of the two in situ formed diastereomers and to visualize the small differences (Supporting Information File 1, Figure S3) in dipolar through space intermolecular interactions in each case, 3D correlation maps were employed. They were displayed carefully so as to ensure the same intensity for the reference intramolecular correlations between NAcTrp H9,9’ with H6 (average distance ≈3.5 Å) and with H8 (average distance ≈4.0 Å) in each of the enantiomers (Figure 1a), enlarging the points of difference in the magnified maps (Figure 1b). Thus (i) guest-H6,H3,H5–host-H5,H66’,H3 interactions are very similar in both enantiomers with guest-H5–host-H3 clearly weaker than the others, and guest-H6/host-H6,6’ stronger in L- than in D-, suggesting that guest-H6,H3 are embedded inside the cavity, guest-H5 is closer to the narrow β-CD rim and L-H6 is closer to it than D-H6. (ii) Guest-H8–host-H3 interactions are equally strong in both enantiomers, stronger than the guest-H8/host-H5,H6,6’ ones, which in turn are stronger in L- than in D-. Moreover, interactions between guest-Me12–host-H3 are strong for both enantiomers (Supporting Information File 1, Figure S3b), suggesting that the *N*-acetyl group is in both cases at the wide secondary opening of β-CD, and L-H8, is closer to the primary opening than D-H8 suggesting a difference in tilting. (iii) Guest-H4–host-H5 interactions are similar in both enantiomers but this of guest-H4–host-H6,6’ is considerably stronger in D- than in L-, while guest-H4–host-H3 interactions are practically absent for both enantiomers, implying that L-H4 is extended further out of the primary side than D-H4. (iv) Guest-H9,9’ and H10 show weak interactions with host-H3 thus they reside mostly closer to the wide opening of the host.

The above interactions detected by NMR suggest that in the aqueous environment the inclusion modes in each diastereomeric complex are very similar but non-identical. D-H4 is located near the primary side of the host, while L-H4 is completely outside (scarcely communicates with the cavity). On the other hand H8 (at a ≈7 Å distance from H4) is at the secondary side in both enantiomers, slightly closer to H5 of the host only in the L-enantiomer. These interactions suggest a common binding model, with the indole part included in the direction H4 to H8 from primary to secondary opening and with the L-enantiomer having its H4 end exposed and its NAc group at the secondary side in contact with CD-H3. A different degree of tilting with respect to the β-CD axis to accommodate the hydrophobic NAc group in the cavity is inferred by the NMR data in each case, thus explaining the small differences observed in solution. However, as the Job plots suggested, the aliphatic part is influenced by a second host molecule presumably via its secondary side. This implies that in solution, host–guest association is possible through additional orientations and stoichiometry, thus the presence of alternative arrangements in low percentage cannot be excluded.

### X-ray crystallography studies

**Figure 2:**
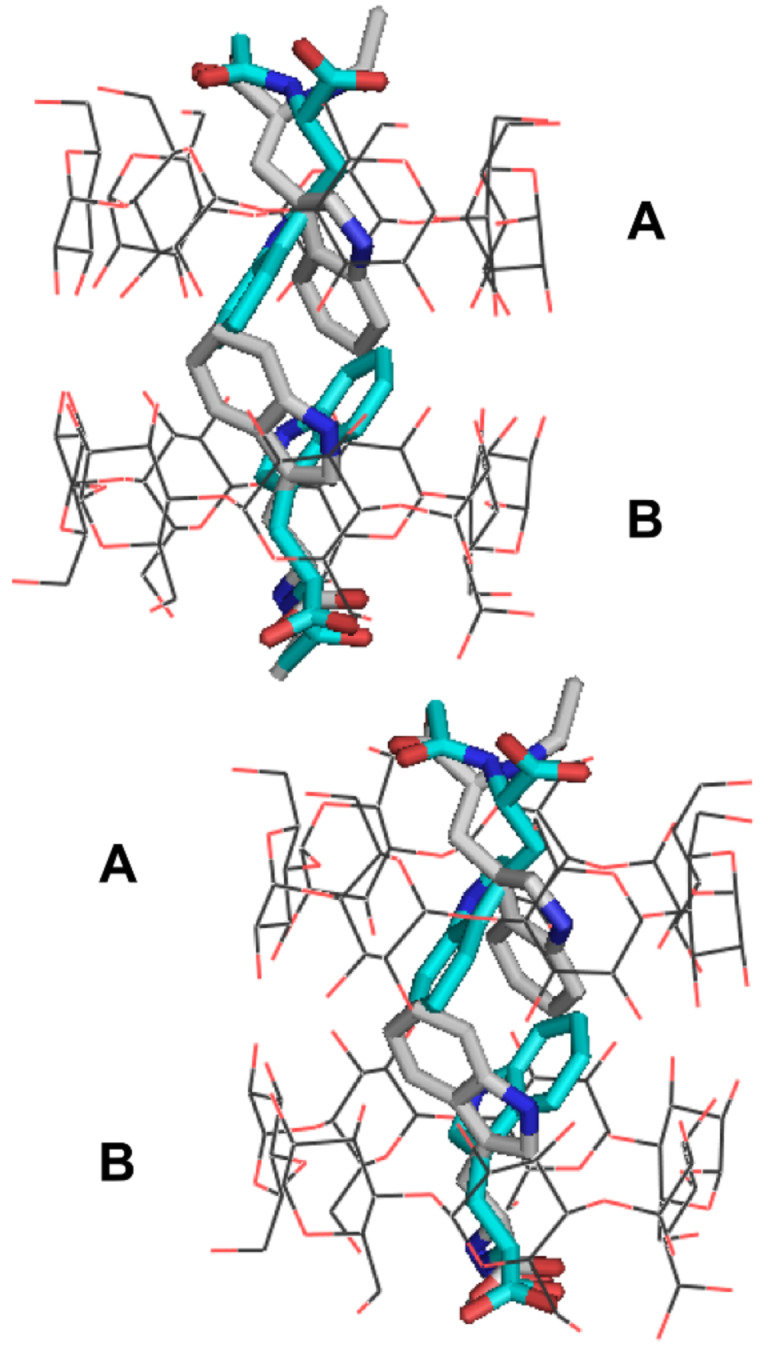
Two dimers of β-CD–L-NAcTrp, stacked along the *a*-axis, are shown. Each β-CD dimer (**A**, **B**) encloses a pair of guest molecules distributed in two orientations, the major (**C** and **D** in cyan) and the minor (**E** and **F**, in grey) respectively.

In the crystalline state, the structure of the inclusion complex of L-NAcTrp in β-CD comprises dimers. The asymmetric unit of the complex contains two crystallographically independent β-CD hosts (**A** and **B**) forming a dimer (Figure 2), in which two guest molecules of L-NAcTrp are enclosed in a head-to-head fashion (host:guest ratio, 1:1). The pair of L-NAcTrp molecules inside the dimer are found in orientational disorder, i.e., the guest exhibits a major orientation, molecules **C** and **D** (occupancy 65%), and a co-existing minor orientation (molecules **E** and **F**, occupancy 35%) in a statistical fashion. The dimers pack along the axis *a* at an angle of 19° thus forming a broken channel (Intermediate packing) [10,27]. The mean distance of the centers of mass of two consecutive β-CD dimers is 5.78 Å. Co-crystallized with each dimer, 21.45 water molecules are found distributed over 36 sites. The water molecules form the usual water networks of H-bonds, one linking the primary and the other the secondary hydroxy groups [28], many of them stabilizing the crystal lattice (structural water molecules).

**Figure 3:**
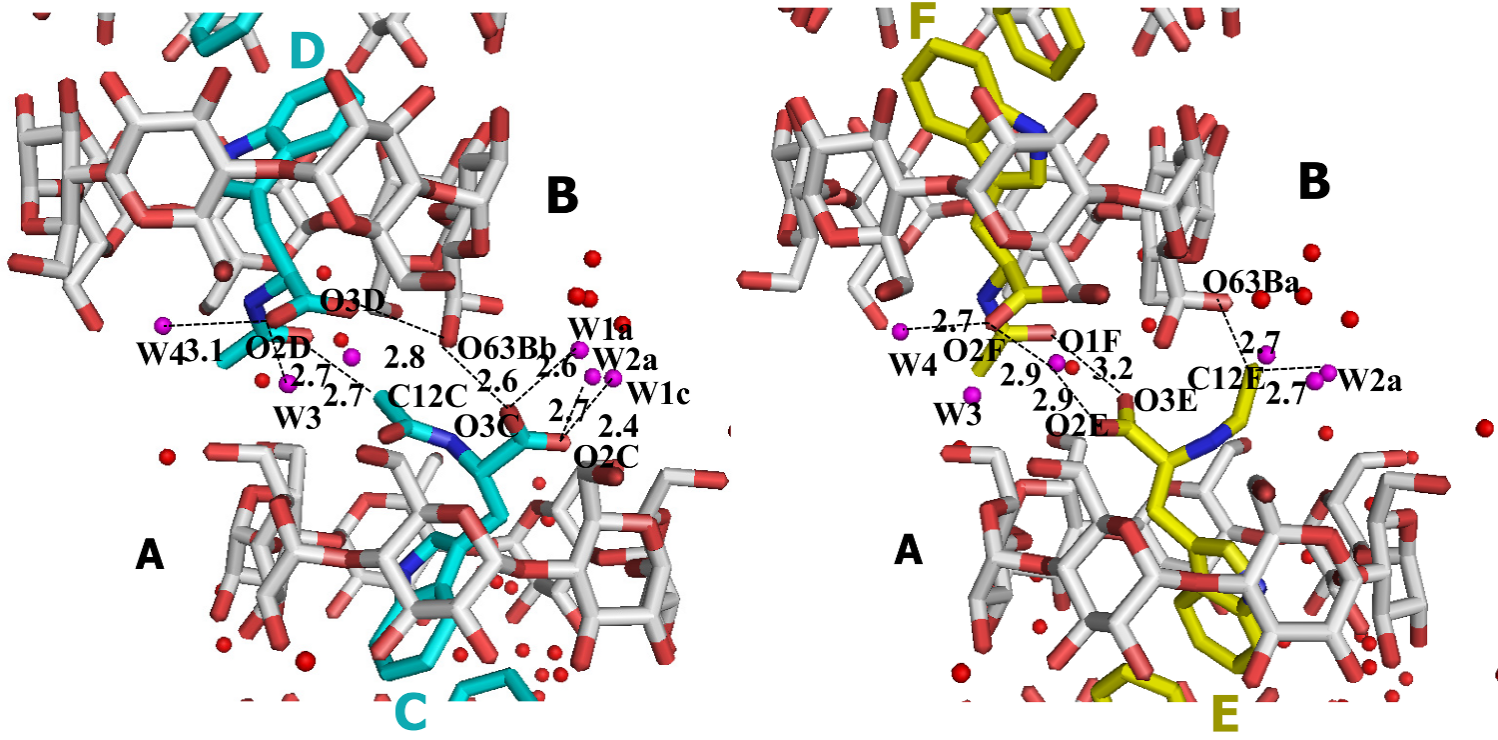
β-CD–L-NAcTrp complex at the interface between two β-CD dimers along the *a*-axis (major orientation guest molecules **C**, **D** in cyan and minor **E**, **F** in yellow). Note that the major guests **C**, **D** interact (a) directly by O1**D** and C12**C** through non-conventional H-bonding and (b) indirectly by mutual H-bonding of O3**C** and O3**D** with the only inward pointing hydroxy Ο63**B**b. Moreover, they are stabilized by the H-bonds of carboxylic oxygen atoms with the structural water molecules OW2a, OW3 and OW4, which interact also with hydroxy groups of the neighbor β-CD hosts. In contrast, the minor guests **E** and **F** interact weakly via the carboxylic O3**E** and acetyl O1**F** atoms and indirectly via a low occupancy water molecule W5, whereas the acetylamino methyl group is exposed to the water molecules in the exterior of the broken channel.

The glucopyranose residues (in ^4^*C*_1_ chair) of both **A** and **B** β-CD have a rather undistorted conformation (Supporting Information File 1, Table S1) (angles between the glycosidic oxygen atoms O-4n similar to these of the regular heptagon, 128.57°, deviations of the O-4n atoms from their mean plane, close to zero). The tilt of the mean glucopyranose planes towards their 7-fold axis are small and close to their average values (7.1 and 7.7°, respectively). As in all β-CD dimeric complexes [28], the macrocycles’ conformation is stabilized by hydrogen bonds connecting (i) intramolecularly, the O-3n and O-2(n+1) atoms of neighboring glucopyranose units (mean 2.73 Å and 2.75 Å for **A** and **B**, respectively, 2.78 Å in hydrated β-CD) and (ii) intermolecularly, the O-3n**A** and O-(8−n)**B** atoms of monomers **A** and **B**, respectively (range of distances 2.7–2.8 Å, Supporting Information File 1, Table S2). At the primary side, only β-CD molecule **B** exhibits disorder of the C-Ο63**Β** bond in two conformations, the major (−)-gauche C-Ο63Βa (occupancy 78%) pointing outward and the minor (+)-gauche C-Ο63Βb (22%) pointing towards the interior of the cavity, the latter interacting with guests **C** and **D** of neighboring dimers (Figure 3a).

The aromatic moieties of both guest orientations maintain the same relative position with the host, their planes interacting in a π···π fashion (Figure 2 and Figure 3) (dihedral angle between the **C** and **D** indole planes, 17.3(1)°, smallest distance 3.5 Å; dihedral angle between **E** and **F**, 14.0(2)°, smallest distance 3.3 Å). The relative positions between major guest **C** and minor **E** enclosed in β-CD **A** are very similar to the relative positions of **D** and **F** enclosed in β-CD **B** (dihedral angles 69.8(6)° and 65.4(7)°, respectively). The dihedral angles between the β-CD mean O-4n planes and indole planes **C** and **D** are the same (57.8(1)° and 57.5(2)°) and very close to the dihedral angles for the minor guests **E** and **F** (58(1) and 61(1)°). The indole nitrogen atoms N1 of guests (**C**, **E**) enclosed in β-CD **A** are almost at the level of the glycosidic oxygen atoms O-4n and close to atoms Ο42Α and O45A, respectively, whereas N1 of the guests (**D**, **F**) in cavity **B** are close to the secondary hydroxy level, apparently in order to optimize the π···π interactions between the indole planes (Figure 2 and Figure 3, Table 1). The above suggest a tight fit of the guest inside the cavity. On the other hand, the aliphatic part of NAcTrp, positioned in the space between dimers, exhibits more freedom: the carboxylic and acetylamino groups of guests **D** and **F** inside β-CD **B** are close and parallel, whereas in β-CD monomer **A** the acetylamino moiety of the major guest **C** is close to the carboxyl group of minor guest **E**, their respective carboxyl and acetylamino groups pointing to opposite directions (Figure 2 and Figure 3). These differences maximize the strong interactions between major guests **C** and **D** (Figure 3, Table 1).

**Table 1:** H-bond distances of β-CD–L-NAcTrp complex: (1) between guest molecules themselves and with the host (2) with water molecules, (3) between structural water molecules and the host.

	Distance (Å)	C_1_-Α_1_···O_2_ (°)	Α_1_···O_2_-C_2_ (°)	Symmetry^i^

1. guest···guest and guest···host interactions				

Major-occupancy guests **C** and **D**				

C12**C**···O1**D**^i^	2.75 (2)	138 (1)	136 (1)	x−1,y,z
Ο3**C**···Ο63**Β**b^i^	2.60 (2)	121 (1)	132 (1)	x−1,y,z
Ν1**C**···Ο42**Α**	3.09 (1)	136.9 (8) 110.4 (8)	106.2 (3) 137.0 (4)	–
O3**D**^i^···O63**B**b^i^	2.81 (3)	154 (2)	94 (1)	x−1,y,z
Ν1**D**^i^···Ο45**Β**^i^	3.38 (2)	138 (2) 90 (2)	101.9 (5) 137.3 (5)	–

Minor-occupancy guests **E** and **F**				

O3**E**···O1**F**^i^	3.18 (8)	132 (1)	131 (1)	x−1,y,z
C12**E**···O63**B**a^i^	2.72 (5)	130 (1)	119 (1)	x−1,y,z
N1**E**···O45**A**	3.06 (6)	147.5 (2) 103.0 (3)	99.8 (3) 142.8 (2)	–

2. guest···water molecules interactions				

Major-occupancy guests **C** and **D**				

Ο3**C**···OW1a	2.61 (2)	114 (1)		
Ο2**C**···OW2a	2.71 (2)	120 (1)		
O2**D**^i^···OW3	2.74 (2)	131 (1)		x−1,y,z
O2**D**^i^···OW4	3.15 (2)	111 (1)		x−1,y,z
Ν2**D**^i^···ΟW4	2.89 (2)	113 (1) 114 (1)		x−1,y,z

Minor-occupancy guests **E** and **F**				

O3**F**^i^···OW5	2.81	96 (1)		x−1,y,z
O2**F**^i^···OW4	2.67	131 (1)		x−1,y,z

3. structural water molecules···host interactions				

OW2a···O61**B**	2.58 (2)		116 (1)	x−1,y−1,z
OW3···O64**A**	2.70 (2)		115 (1)	x,y+1,z
OW3···O67**A**	2.68 (2)		109 (1)	x,y,z
OW4···O64**A**	2.89 (2)		105 (1)	x,y+1,z

^i^Atomic position equivalent by symmetry; a or b on atom names refer to different disordered positions of the atom.

**Figure 4:**
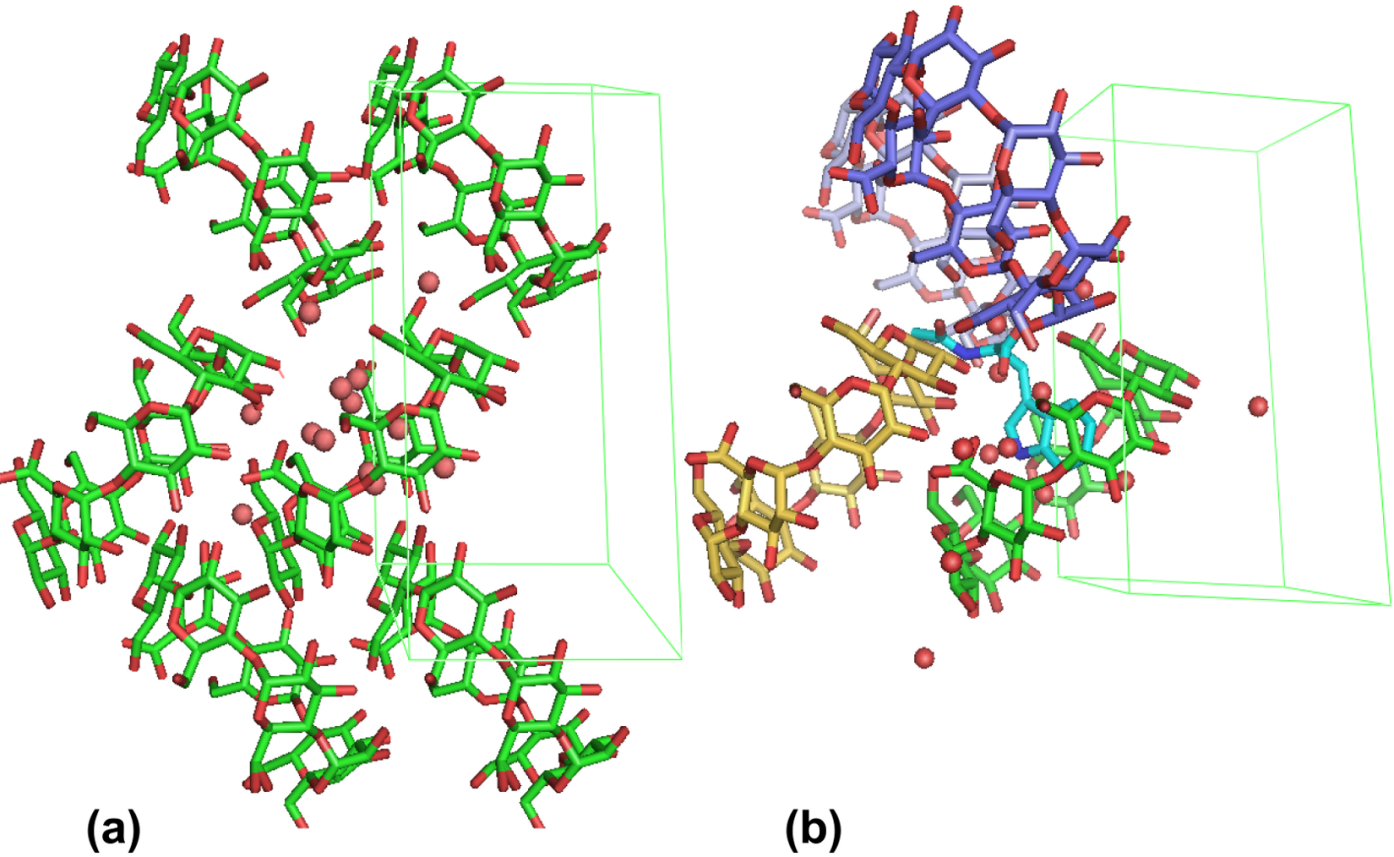
“β-CD–D-NAcTrp” structure. (a) The herring bone packing of β-CD along the *c*-axis; (b) The guest (cyan) can be accommodated in the “β-CD–D-NAcTrp” monomeric structure as indicated by molecular modeling studies (water molecules of the asymmetric unit are shown as spheres).

Numerous trials to crystallize the inclusion complex of β-CD with D-NAcTrp have failed to give anything but hydrated β-CD crystals [29], as described in detail in the experimental section, however, some crystals were grown after hydrothermal treatment of the solution (65 °C for duration of 6 days) [30–31]. The structure of the latter could not be solved by isomorphous replacement (using the coordinates of β-CD–glutaric acid complex [32], that is isomorphous to hydrated β-CD [29]. This was an indication that the structure should be quite different from hydrated β-CD. However, no guest could be located during the refinement and the present structure (henceforth “β-CD–D-NAcTrp”) was refined as a β-CD–water complex (Table 2). “β-CD–D-NAcTrp” exhibits the “herringbone” packing of the β-CD monomers (Figure 4) as the hydrated β-CD structures reported so far [29,33–35], as well as several monomeric β-CD complexes [32,36–37]. The conformation of the β-CD macrocycle (Supporting Information File 1, Table S3) is similar to the monomeric β-CD structures [29], but more distorted than in the dimeric β-CD–L-NAcTrp complex: The glucopyranose residues adopt the regular ^4^*C*_1_ chair conformation, but the angles between them deviate from the angle of the regular heptagon and the tilt of their average planes towards the 7-fold β-CD axis varies between 5.0 and 25.8°. At the primary side, two hydroxy groups (O61 and O65) point towards the interior of the cavity and two exhibit two-way disorder of the C-Ο63 and C-Ο67 bonds.

**Table 2:** Details of crystal and structure refinement data of the complexes. β-CD–L-NAcTrp and the β-CD–H2O (“β-CD–D-NAcTrp”).

	β-CD–L-NAcTrp	“β-CD–D-NAcTrp”

molecular formula	C_110_H_113.6_N_4_O_97.45_	C_42_H_49_O_46.76_
formula weight	3050.85	1301.98
temperature	100 K	100 K
radiation/wavelength	0.8015	0.80
space group	*P*1	*P*21
*a*, α	17.760(6) Å, 102.77(3)°	14.970(5) Å
*b*, β	15.158(6) Å, 99.35(4)°	10.175(2) Å, 112.37(1)°
*c*, γ	15.237(7) Å, 113.00(3)°	21.298 (4) Å
volume/Z	3538(3) Å^3^/1	3000(1) Å^3^/2
density (calculated)	1.432 mg/m^3^	1.436 mg/m^3^
2θ range for data collection	9.28–57.74°	3.0–47.16°
index ranges	0 < h < 21, −18 < k < 16, −18 < l < 17	−14 < h < 14, −10 < k < 10, −21 < l < 21
reflections collected/unique	26878/11889	9397/5511
solution method	isomorphous replacement	molecular replacement
refinement method	full-matrix least-squares on F^2^	full-matrix least-squares on F^2^
data[Fo > 4σ(Fo)]/restraints/parameters	11771/184/1983	5516/683/801
goodness-of-fit on F^2^	1.076	1.065
R indices [F_o_>4σ(F_o_)]	R1 = 0.0609, wR2 = 0.1663	R1 = 0.0815, wR2 = 0.1989
R indices (all data)	R1 = 0.0613, wR2 = 0.1674	R1 = 0.0815, wR2 = 0.1989
largest diff. peak and hole	0.87 and −0.52	0.59 and −0.62

Comparison of the “β-CD–D-NAcTrp” structure to this of hydrated β-CD [29] pinpoints the difficulty of solving the structure as an isomorph. It can be seen (after the appropriate transformation of coordinates due to different origin and axes; Supporting Information File 1, Figures S4 and S5) that the hydrated β-CD macrocycle does not superpose exactly in the lattice of “β-CD–D-NAcTrp”, which may render the two structures not quite isomorphous. It is worth noting that many of the hydrated β-CD structures [29,33–35], as well as several monomeric β-CD complexes [32,36–37] are determined in lattices with different origin or interchanged crystallographic axes or even inverse coordinates (Supporting Information File 1, Figure S4). Further, by superposition of one glucopyranose unit of “β-CD–D-NAcTrp” to the equivalent unit of hydrated β-CD [29] the difference in coordinates of the two structures is more apparent (Supporting Information File 1, Figure S6). In contrast, the same kind of superposition applied to monomeric structures mentioned above shows that they superpose completely on hydrated β-CD.

**Figure 5:**
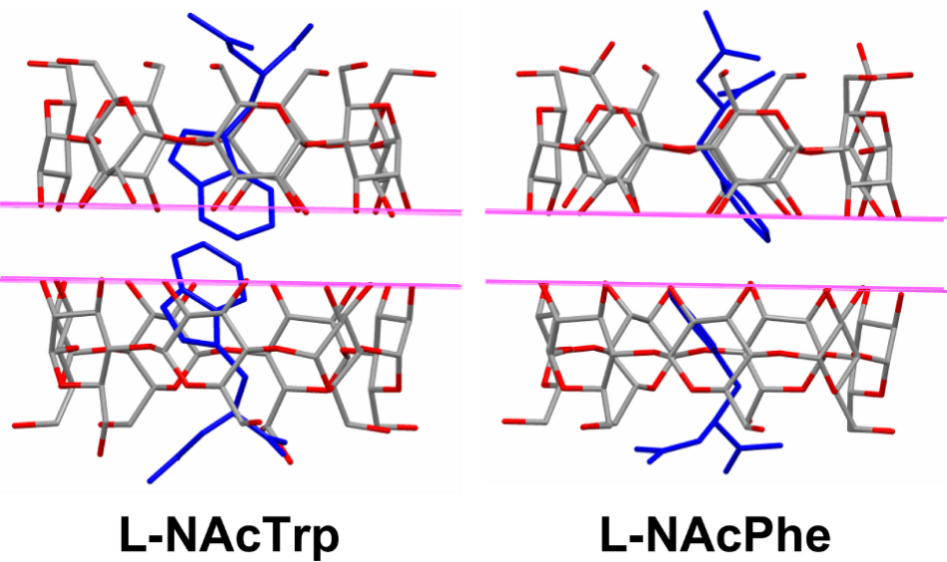
L-NAcTrp and L-NAcPhe in β-CD dimers (the lines indicate the levels of the O2 and O3 secondary hydroxy groups.

Although the NMR results have shown that β-CD forms complexes with both L- and D-NAcTrp in aqueous solution at room temperature, it was not possible to crystallize the β-CD–D-NAcTrp complex. In contrast, the β-CD complexes of both enantiomers of *N*-acetylphenylalanine (NAcPhe) have been determined [18] and they are isomorphous with β-CD–L-NAcTrp. Although the isomorphous complexes of L-NAcPhe and D-NAcPhe exhibit identical packing of the β-CD dimers, the relative stability of the guest molecules enclosed in them is controlled by subtle changes in the guest positioning. L-NAcPhe is highly disordered even at 20 K probably due to very weak non-polar and polar interactions, whereas D-NAcPhe is highly ordered, although the non-polar interactions between the phenyl moieties are also weak. Its stability is gained by the *N*-acetyl group of one D-NAcPhe guest, which rotates and “hides” inside the dimer cavity [18] (probably because of unfavourable exposure to the aqueous environment). Similarly, β-CD–L-NAcTrp is also more stable than β-CD–L-NAcPhe due to the larger side chain of the guest. L-NAcPhe is shorter than in L-NAcTrp, which has two consequences for the stability of the complex (a) no strong π···π interactions at 3.5 Å can be established in the middle of the β-CD dimer as in L-NAcTrp (Figure 5); (b) the aliphatic moieties of β-CD–L-NAcPhe protruding from the primary sides between dimers do not interact directly or even indirectly via β-CD hydroxy groups along the channels, as in the L-NAcTrp complex. Modeling the possibility of formation of a dimer β-CD–D-NAcTrp complex by energy minimization of the interactions of D-NAcTrp inside the β-CD dimer (as determined in the β-CD–L-NAcTrp structure) revealed a complex similar to β-CD–L-NAcTrp (Supporting Information File 1, Figure S7). The positioning of the **D**-indole groups is very similar to these of the L-enantiomer (closest distance 3.46 Å between the aromatic planes). The approaching aliphatic moieties between two β-CD dimers along the channel could be stabilized possibly by an inward pointing hydroxy group Ο63Βb of β-CD (assuming that the β-CD host remains unchanged), which H-bonds to the carboxylic oxygen atom of the **D** guest and the acetyl O1 atom of the **C** guest, however, the acetyl methyl group of **C** is exposed to the water environment. “Hiding” of the latter group inside the cavity, as in the case of the β-CD–D-NAcPhe complex, is not possible due to the bulkier indole group of D-NAcTrp that fills the cavity. This unfavorable environment might be a factor that forbids the formation of a β-CD–D-NAcTrp dimer structure.

The difficulty in crystallizing the β-CD–D-NAcTrp may arise from a higher free energy barrier of crystal nucleation compared to other competing processes in solution at room temperature, but under the higher temperature and pressure conditions of the hydrothermal cell the presence of D-NAcTrp or of the complex β-CD–D-NAcTrp may influence the initial crystal nuclei which eventually lead to the grown crystals and differentiates them slight from hydrated β-CD. It is worth noting that hydrothermal treatment in crystallization trials has yielded uncommon structures such as, novel packing of β-CD–ethanol crystals [31] during trials to crystallize the β-CD–*N*-(1-adamantyl)salicylaldimine complex in ethanol, novel association of β-CD monomers in structures of β-CD complexes, e.g., with 4-pyridinealdazine [30], polyethylene glycol [38] or adamantane [39].

## Conclusion

This work has been focused on the ability of β-CD to discriminate between the enantiomers of *N*-acetyltryptophan. NMR studies in aqueous solution show that both enantiomers form similar, but not identical complexes with β-CD. L-NAcTrp induces larger shifts of β-CD cavity protons, suggesting stronger binding. For both enantiomers the prevailing complexation mode involves insertion in the cavity with the *N*-acetyl group in the secondary side and the indole moiety exiting the primary side, more exposed in L- than in D-NAcTrp. The tendency of the *N*-acetyl group to hide in the cavity is considered as the major cause for the differences between the two complexes that also results in somewhat folded NAcTrp structures, compared to the conformation observed in the crystal. In addition, both complexes are in contact with a second β-CD molecule suggesting presence of higher stoichiometries and possibility of different inclusion modes at low concentration. Overall, the orientation of both enantiomeric guests with respect to the macrocycle in the solution structures is opposite to the orientation of L-NAcTrp in ther crystal.

On the other hand, only the complex β-CD–L-NAcTrp crystallizes readily forming a dimeric complex (two host and two guest molecules) packed in broken channels, isomorphous to the known β-CD complexes of the NAcPhe enantiomers. Numerous crystallization trials failed to produce crystals of the β-CD–D-NAcTrp complex yielding only hydrated β-CD crystals. The fact that β-CD–D-NAcTrp could not be crystallized in dimers as the β-CD–L-NAcTrp might be due to destabilization of the interface between dimers, because of exposure of the acetyl group to the water environment of the exterior and the inability to “hide” in the cavity, due to the bulky indole group occupying it. Trials to employ more energetic crystallization conditions resulted in crystals of a slightly different structure than hydrated β-CD crystals. The disagreement between solution and crystal structure in terms of complex formation and orientation/conformation of the guest indicates that the lattice forces and organization in the crystal prevail by far over the soft host–guest contacts established in solution and determines the final orientation of the guest inside the host and the formation of the crystals per se.

## Experimental

### Materials and methods

*N*-Acetyl-L-tryptophan (L-NAcTrp), *N*-acetyl-D-tryptophan (D-NAcTrp) and β-CD were obtained from Sigma-Aldrich. Deuterium oxide was a product of Deutero GmbH.

#### NMR spectroscopy

The spectra were carried out on a 500 MHz Bruker Avance instrument at 300 K using a BBI probe, the library pulse sequences and 300 ms mixing time for the 2D ROESY runs. The compounds were dissolved in unbuffered D_2_O. The data was processed with Topspin.

#### X-ray crystallography

**Crystallisation of β-CD–L-NAcTrp.** In an aqueous solution of β-CD (6 mM) an equimolar quantity of L-NAcTrp was added and stirred for an hour until the solution became clear, which indicated formation of a complex. Then the solution was placed in an incubator at 23 °C, where by slow evaporation of the solvent, single crystals appropriate for X-ray data collection were obtained. The crystals had a diamond shape and a slightly pink color.

**Crystallisation trials of β-CD–D-NAcTrp***.* Trials to crystallize the complex of β-CD with D-NAcTrp under various conditions, including the above, did not result to single crystals of the complex. D-NAcTrp in the presence of β-CD (6 mΜ) at 50–60 °C, required a small quantity of ethanol in order to obtain a clear solution, from which crystals of hydrated native β-CD precipitated. This was proved from data collection from several crystals and structure determination based on isomorphous replacement using the coordinates of the β-CD–glutaric acid complex [32], which is isomorphous to hydrated β-CD [29]. Use of racemic mixtures of NAcTrp produced also native β-CD crystals. However, use of a hydrothermal cell [30–31], in which β-CD (0.050 mM) and D-NAcTrp (0.025 mM) were placed in 2 mL of water and left at 65 °C for 5–7 days, produced crystals that could not be refined by isomorphous replacement using the coordinates of hydrated β-CD or other isomorphous crystals, as above.

**Structure determination.** Low temperature X-ray data were collected at synchrotron radiation light sources. A single crystal, covered with a drop of paraffin oil, was mounted on a hair fiber loop and was instantly frozen to 100 K. Crystal data and analysis details are given in Table 2.

**β-CD–L-NAcTrp.** Data of the β-CD–L-NAcTrp complex were collected at the beamline X13 of EMBL at DESY, Hamburg, by the oscillation method using a CCD of 165 mm radius detector. The DENZO and SCALEPACK [40] software were used for data processing and scaling, respectively. The unit cell parameters and their esds were determined by the least square method from the high resolution frames of the collected data. The structure was solved by the isomorphous replacement method using the host coordinates of the β-CD–1,12-dodecanodioic acid complex [28]. The structure solution and the refinement were carried out with the SHELXL97 program [41]. The coordinates of the guest and solvent atoms were determined by successive cycles of difference maps and refinement. The non-hydrogen β-CD atoms and the oxygen atoms of the co-crystallized water molecules were treated anisotropically. Hydrogen atoms were placed at idealized positions and refined by the riding model (UH = 1.25 UC). The refinement of the structure, by full matrix least squares, converged to R1 = 0.0609, wR2 = 0.1663 and Goodness-of-fit = 1.076, for Fo > 4σ(Fo). Refinement details appear in CCDC 1531988. The structures were rendered in PyMOL [42].

**“β-CD–D-NAcTrp”.** Diffraction data were collected at the X06DA beamline, Swiss Light Source, Paul Scherrer Institut, Villigen, Switzerland. The XDS [43] software package was used to reduce data and determine the unit cell parameters and space group, which were the same as hydrated β-CD. Trials to use isomorphous replacement (using the coordinates of β-CD–glutaric acid complex [32], which is isomorphous to hydrated β-CD [29], to refine the structure was unsuccessful (vide supra). The structure was solved finally by molecular replacement methods [44] using the β-CD–glutaric acid complex coordinates. The refinement was carried out with the same strategy as in β-CD–L-NAcTrp complex. Early in the refinement numerous peaks appeared mainly at the primary hydroxy side of the cavity. Some were at bonding distances with each other, but by introducing the strongest of them as water molecules into the refinement did not result in a model of the guest (Table 2). Refinement details appear in CCDC 1531987. The structures were rendered in PyMOL [42].

#### Molecular modeling

The molecular models of D-NAcTrp complexes were based (a) on the geometry of the major orientation of β-CD–L-NAcTrp by changing the chirality of the Cα atom and (b) on β-CD non-hydrogen atoms of the corresponding lattice. To relieve steric clashes, restrained energy minimization of D-NAcTrp have been performed, while non-hydrogen atoms of β-CD are kept fixed in space. The XLEaP module of the AMBER 16 suite [45] was used and the GAFF parameters were applied to the β-CD molecules with AM1-BCC atomic charges using the Antechamber module [46], while the ff99SB parameters were employed for NAcTrp. Restraint energy minimizations in implicit solvent were performed for 1,000 steps using a pairwise generalized Born model [47], while all β-CD non-hydrogen atoms were kept fixed in space using harmonic restraints of 10 kcal/mol Å^2^. For the “β-CD–D-NAcTrp” complex, the indole moiety was placed inside the β-CD cavity with the aliphatic part protruding from its primary side towards the empty space formed by three neighboring β-CD monomers of the lattice (Figure 4b), whereas for the β-CD–D-NAcTrp dimer model the crystallographic coordinates of the β-CD–L-NAcTrp dimer were employed after changing the chirality of the L-NAcTrp Cα atom only to generate the D-NAcTrp guest molecule.

## Supporting Information

File 1 Experimental data containing geometry data of the β-CD hosts; H-bonding interactions in the β-CD dimer; NMR data (Job plots and 2D maps of the observed dipolar interactions); packing, origin selection and comparison of monomeric β-CD complexes; modeling results of D-NAcTrp/β-CD.
