# Supplementary material for: Molecular recognition of N-acetyltryptophan enantiomers by β-cyclodextrin
Source: Beilstein J Org Chem. 2017 Aug 9;13:1572–82. doi: 10.3762/bjoc.13.157 (PMC5564276; doi:10.3762/bjoc.13.157)
Supplement: File 1 — Experimental data containing geometry data of the β-CD hosts; H-bonding interactions in the β-CD dimer; NMR data (Job plots and 2D maps of the observed dipolar interactions); packing, origin selection and comparison of monomeric β-CD complexes; modeling results of D-NAcTrp/β-CD. [file Beilstein_J_Org_Chem-13-1572-s001.pdf]

Supporting Information

for

Molecular recognition of *N*-acetyltryptophan enantiomers by β-cyclodextrin

Spyros D. Chatziefthimiou, Mario Inclán, Petros Giastas, Athanasios Papakyriakou, Konstantina Yannakopoulou* and Irene M. Mavridis*

Address: Institute of Nanoscience & Nanotechnology, National Center for Scientific Research “Demokritos”, Patriarchou Gregoriou E’ & Neapoleos 27, Aghia Paraskevi Attikis, 15310 Greece

Email: Irene M. Mavridis - e.mavridis@inn.demokritos.gr

*Corresponding author

Experimental data containing geometry data of the β-CD hosts; H-bonding interactions in the β-CD dimer; NMR data (Job plots and 2D maps of the observed dipolar interactions); packing, origin selection and comparison of monomeric β-CD complexes; modeling results of D-NAcTrp/β-CD

Contents

1. Geometry of the β-CD Complexes

Tables S1–S3.

2. Determination of the host/guest ratio by Job plots & 2D ROESY NMR maps of the complexes overlayed

Figures S1–S3

3. Packing and origin selection of monomeric β-CD complexes

Figure S4.

4. Comparison of the structure of hydrated β-CD with “β-CD–D-NAcTrp” and other monomeric structures

Figures S5 and S6

5. Energy minimization of D-NAcTrp inside the dimer structure of the β-CD–L-NAcTrp complex.

Figure S7.

Table S1: Geometrical parameters of the β-CD in the β-CD–L-NAcTrp complex

Glucose	D[a] (Å)	φ[b] (°)	d[c] (Å)	Tilt Angles[d] (°)	D3[e] (Å)	Tortion Angles(°) $O5_n$-$C5_n$-$C6_n$-$O6_n$
**β-CD Molecule A**						
**G1**	4.294 (4)	128.0 (1)	-0.036 (3)	5.0 (2)	2.746 (6)	-67.1 (5)
**G2**	4.300 (5)	128.1 (1)	0.005 (3)	9.4 (2)	2.718 (5)	-61.2 (6)
**G3**	4.358 (6)	128.78 (9)	0.021 (2)	5.2 (1)	2.666 (5)	-65.7 (5)
**G4**	4.331 (5)	129.9 (1)	-0.011 (3)	7.2 (2)	2.727 (5)	-58.5 (5)
**G5**	4.280 (5)	126.8 (1)	-0.011 (3)	7.8 (2)	2.756 (5)	-59.1 (6)
**G6**	4.374 (6)	128.5 (1)	0.002 (2)	5.6 (1)	2.720 (5)	-66.5 (4)
**G7**	4.316 (5)	129.9 (1)	0.030 (2)	9.8 (2)	2.795 (5)	-69.3 (7)
**β-CD Molecule B**						
**G1**	4.239 (5)	131.2 (1)	-0.014 (2)	11.9 (1)	2.767 (5)	-69.1 (6)
**G2**	4.324 (4)	123.9 (1)	0.006 (3)	8.6 (1)	2.765 (6)	-65.3 (5)
**G3**	4.317 (5)	128.5 (1)	-0.006 (3)	9.1 (2)	2.804 (5)	45.4 (1.2)<br>-77.3 (7)
**G4**	4.410 (5)	132.3 (1)	0.004 (3)	5.7 (2)	2.748 (5)	-63.8 (5)
**G5**	4.280 (5)	128.3 (1)	0.007 (3)	5.4 (2)	2.656 (6)	-55.0 (6)
**G6**	4.273 (5)	123.3 (1)	-0.020 (3)	6.4 (1)	2.732 (5)	62.2 (6)
**G7**	4.467 (6)	132.4 (1)	0.023 (2)	6.7 (2)	2.771 5)	-69.4 (5)

[a] O-4*n*···O-4(*n*+1); [b] O-4(*n*-1)···O-4*n*···O-4(*n*+1) angles. [c] Deviations (Å) from the least-squares optimum plane of O-4*n* atoms. [d] Tilt angles between the optimum O-4*n* plane and the mean planes through atoms O-4(*n*-1), C-1*n*, C-4*n*, O-4*n*. [e] Intramolecular H-bonds between O-3*n*···O-2(*n*+1). [f] Orientation of the C-6*n*-O-6*n* bond.

Table S2: Intermolecular hydrogen bond distances between the O3 atoms of the β-CDs in the dimer of the β-CD–L-NAcTrp complex

O_A···O_B	Distance (Å)	C_A-O_A···O_B (o)	O_A···O_B-C_B (°)
**O31A···O37B**	2.713 (5)	117.7 (3)	118.8 (3)
**O32A···O36B**	2.802 (5)	120.1 (3)	119.0 (3)
**O33A···O35B**	2.754 (5)	118.5 (3)	119.0 (3)
**O34A···O34B**	2.735 (5)	119.6 (3)	119.3 (3)
**O35A···O33B**	2.778 (5)	120.9 (3)	115.6 (2)
**O36A···O32B**	2.730 (5)	121.3 (3)	111.9 (3)
**O37A···O31B**	2.780 (5)	117.1 (3)	119.3 (3)

Table S3: Geometrical parameters of the β-CD in the β-CD–D-NAcTrp complex.

Gn	D[a] (Å)	Φ[b] (˚)	D[c](Å)	D_3[d](Å)	Tilt angles[e] (˚)	Torsion Angles(°) $O5_n$–$C5_n$–$C6_n$–$O6_n$
**β-CD**						
**G1**	4.308(5)	126.6(1)	0.044(3)	2.914(6)	25.8(3)	71(1)
**G2**	4.445(5)	126.8(1)	-0.203(3)	2.914(6)	9.0(2)	-63.5(5)
**G3**	4.454(5)	132.5(1)	-0.002(3)	2.785(6)	10.4(3)	-63.0(6)<br>-44(2)
**G4**	4.223(5)	127.1(1)	0.262(3)	2.780(5)	11.8(4)	-72.3(5)
**G5**	4.323(6)	124.6(1)	-0.159(3)	2.901(6)	20.1(3)	65.0(6)
**G6**	4.522(6)	133.1(1)	-0.163(4)	2.864(6)	Out 5.0(2)	-64.8(5)
**G7**	4.352(6)	127.4(1)	0.220(3)	2.936(7)	18.7(4)	-65.1(9)<br>56(1)

[a]O-4*n*⋯O-4(*n*+1); [b]O-4(*n*-1)⋯O-4*n*⋯O-4(*n*+1) angles. [c]Deviations (Å) from the least-squares optimum plane of O-4*n* atoms. [d] Intramolecular H-bonds between O-3*n*⋯O-2(*n*+1). [e]Tilt angles between the optimum O-4*n* plane and the mean planes through atoms O-4(*n*-1), C-1*n*, C-4*n*, O-4*n*; [f] Orientation of the C-6*n*-O-6*n* bond.

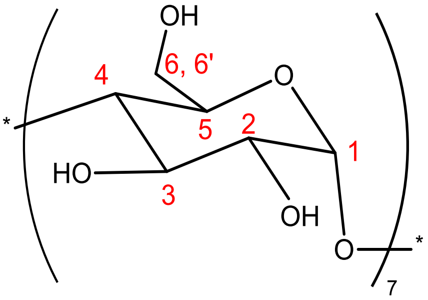

β-CD

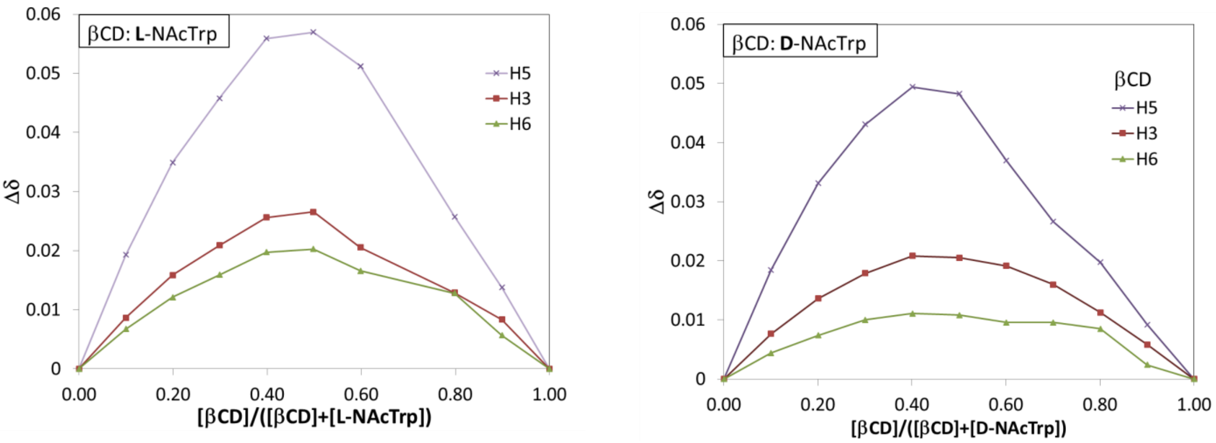

Figure S1: Job plots of ^{1}H NMR signals of β-CD upon interaction with L-NAcTrp (left) and D-NAcTrp (right).

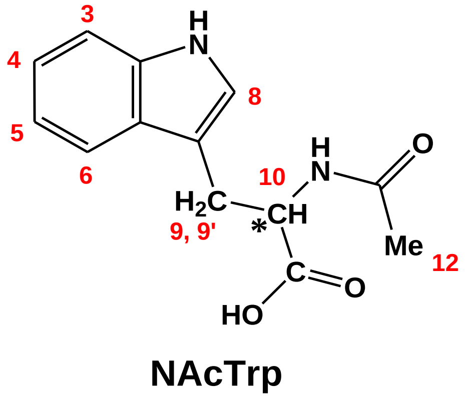

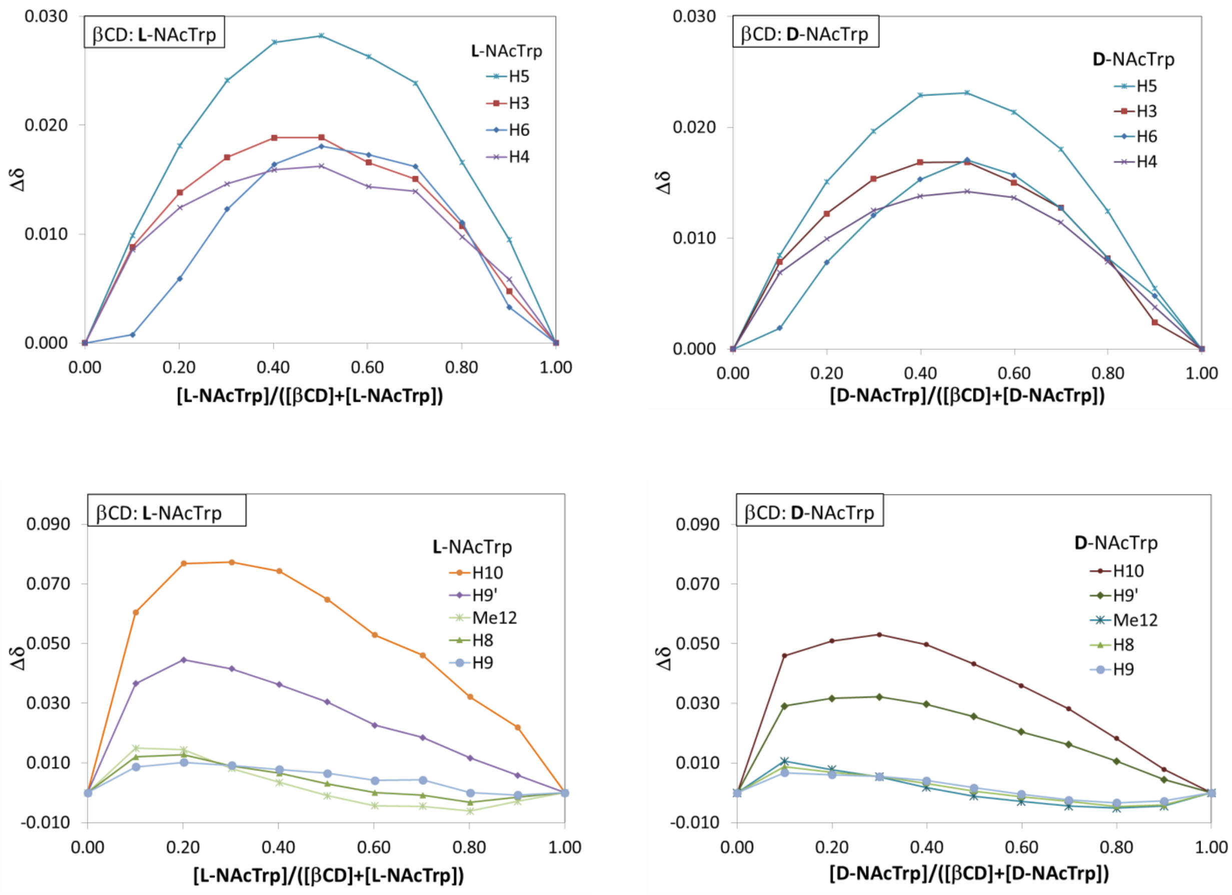

Figure S2: Job plots of ^{1}H NMR signals of L-NAcTrp (left) and D-NAcTrp (right) upon interaction with β-CD.

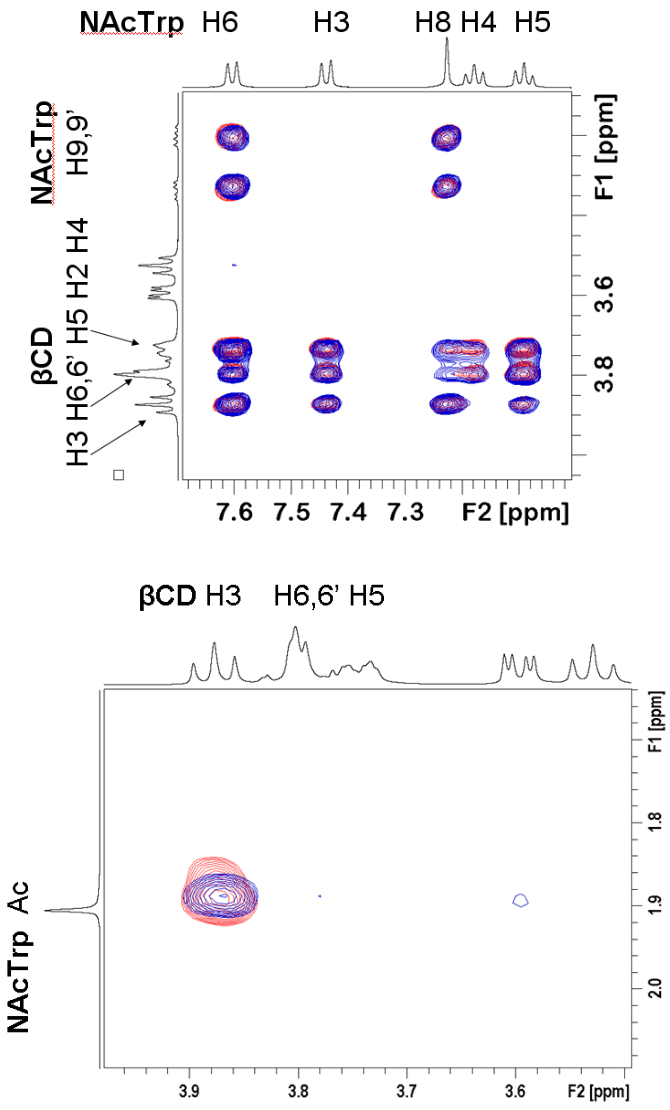

(a)

(b)

Figure S3: 2D maps of the observed dipolar, through space host–guest interactions (D-NAcTrp = red contours; L-NAcTrp = blue contours) overlayed so that the strong intramolecular cross-peaks between H9,9' with H6 and H8 in each NAcTrp enantiomer are of equal intensity. (a) The differences observed concern through space interactions between guest protons H8 and H4 and host H6,6' and H5 only, all other interactions being practically identical. (b) The acetate group shows through space dipolar interaction with β-CD H3 only, i.e., the NAc group is located exclusively near the wider secondary side of the host.

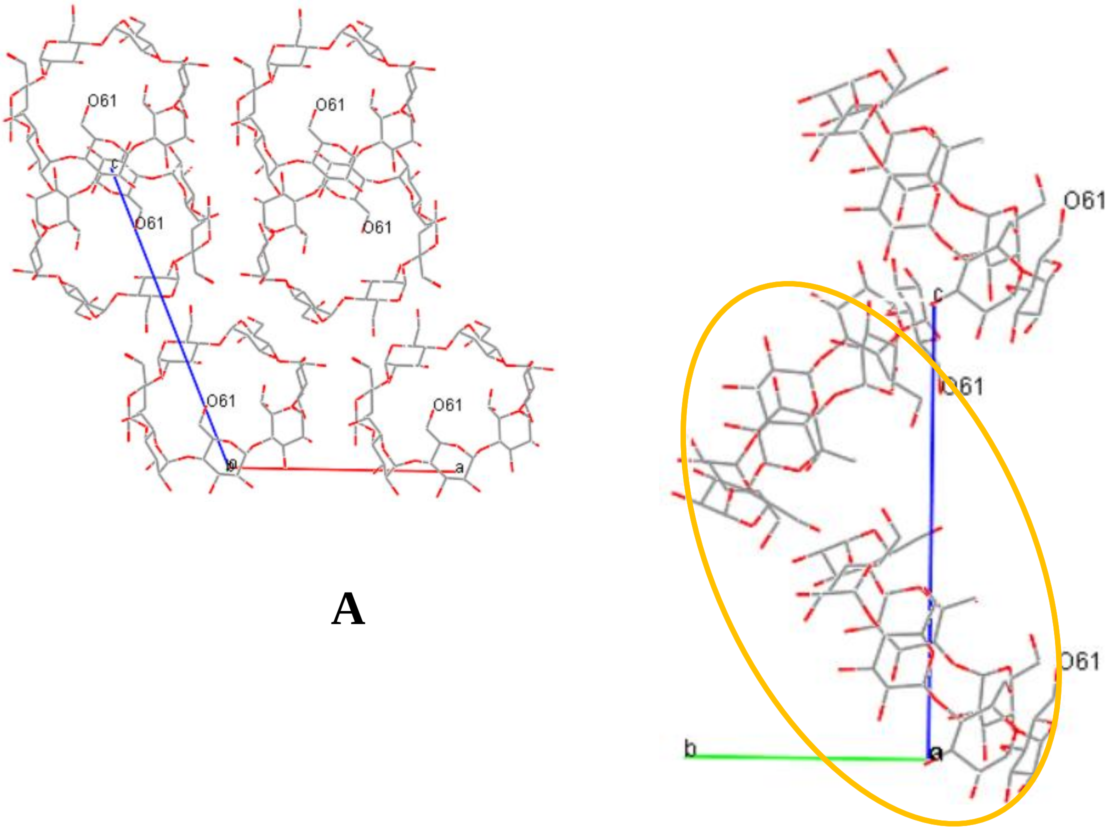
O61
O61
O61
O61
O61
O61
O61
O61
c
a
b

A

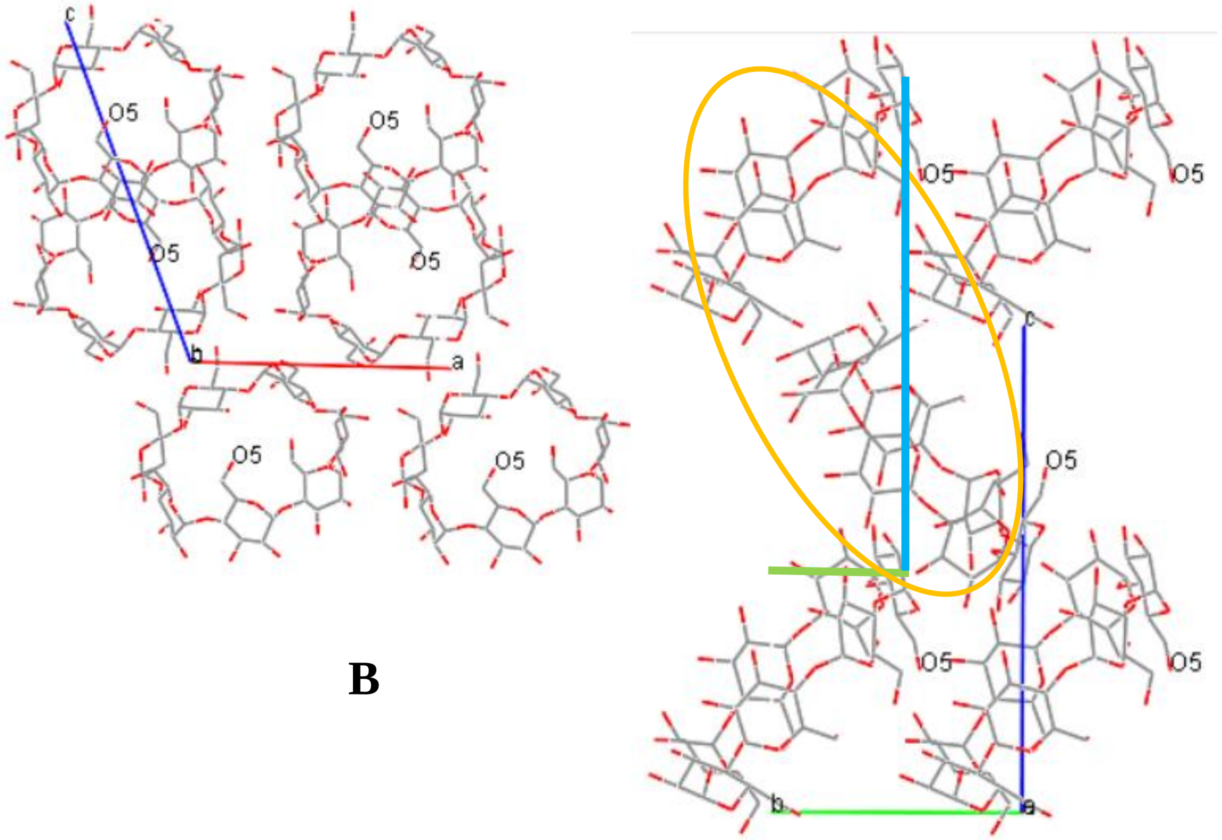
O5
O5
O5
O5
O5
O5
O5
O5
O5
O5
O5
c
a
b

B

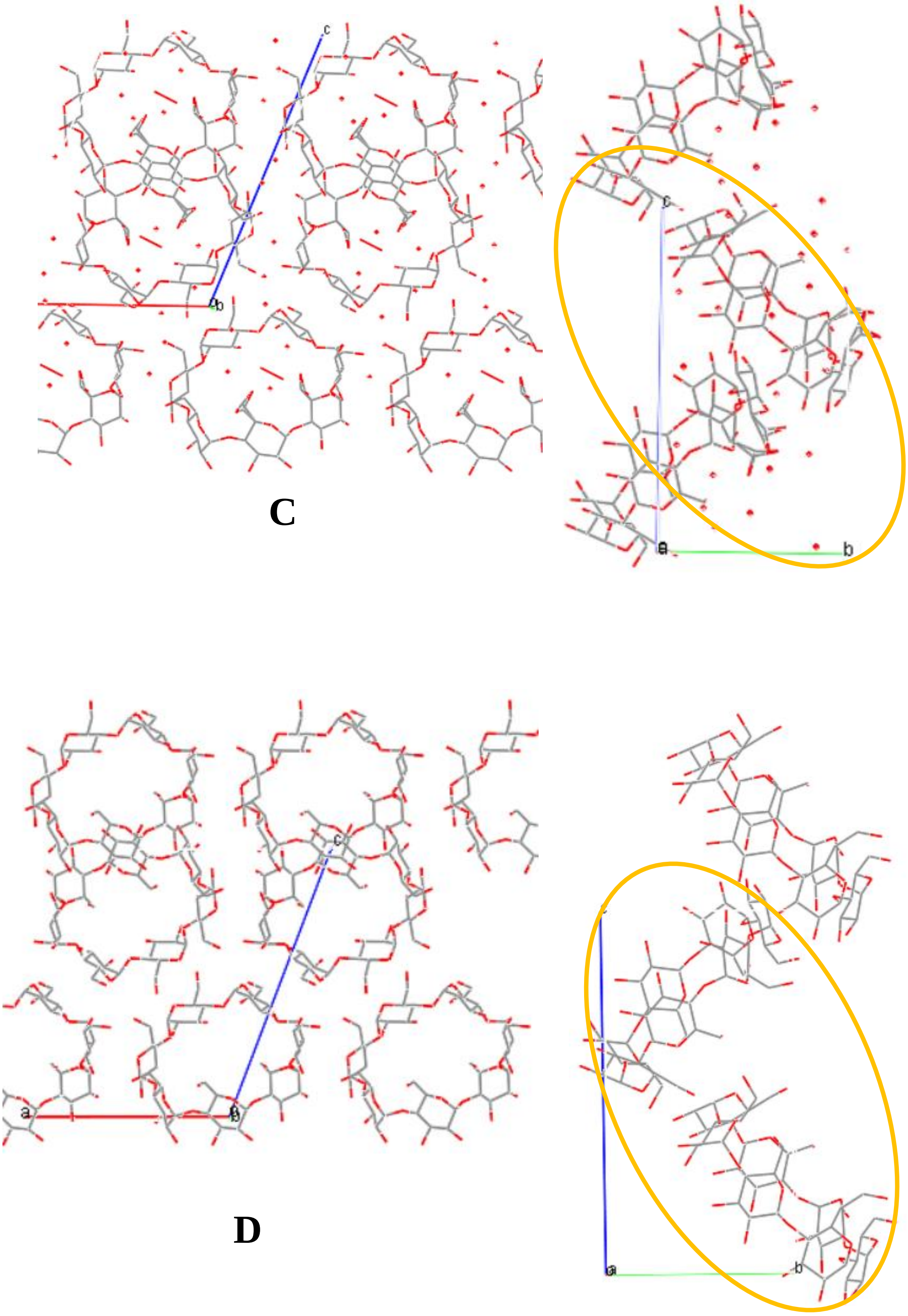

Figure S4: Packing[1] of monomeric β-CD complexes (*left*, in the ac plane and *right* in the bc plane). (A) “β-CD–D-NAcTrp”; (B) β-CD–Hydrate clathrate (BUVSEQ01)[2] after transformation of axes from **abc** to **c(-b)a**. Note that if the origin in BUVSEQ01 is moved by 1/2**c** and by 0.429**b** (new axes in bold), its coordinates would almost superpose with these of “β-CD–D-NAcTrp”; (C) β-CD–Hydrate (OXAGUQ)[3]: The structure is the same as BUVSEQ01); (D) β-CD–Hydrate (GAGPOA)[4]. The structure superposes on “β-CD–D-NAcTrp”after invertion of its coordinates.

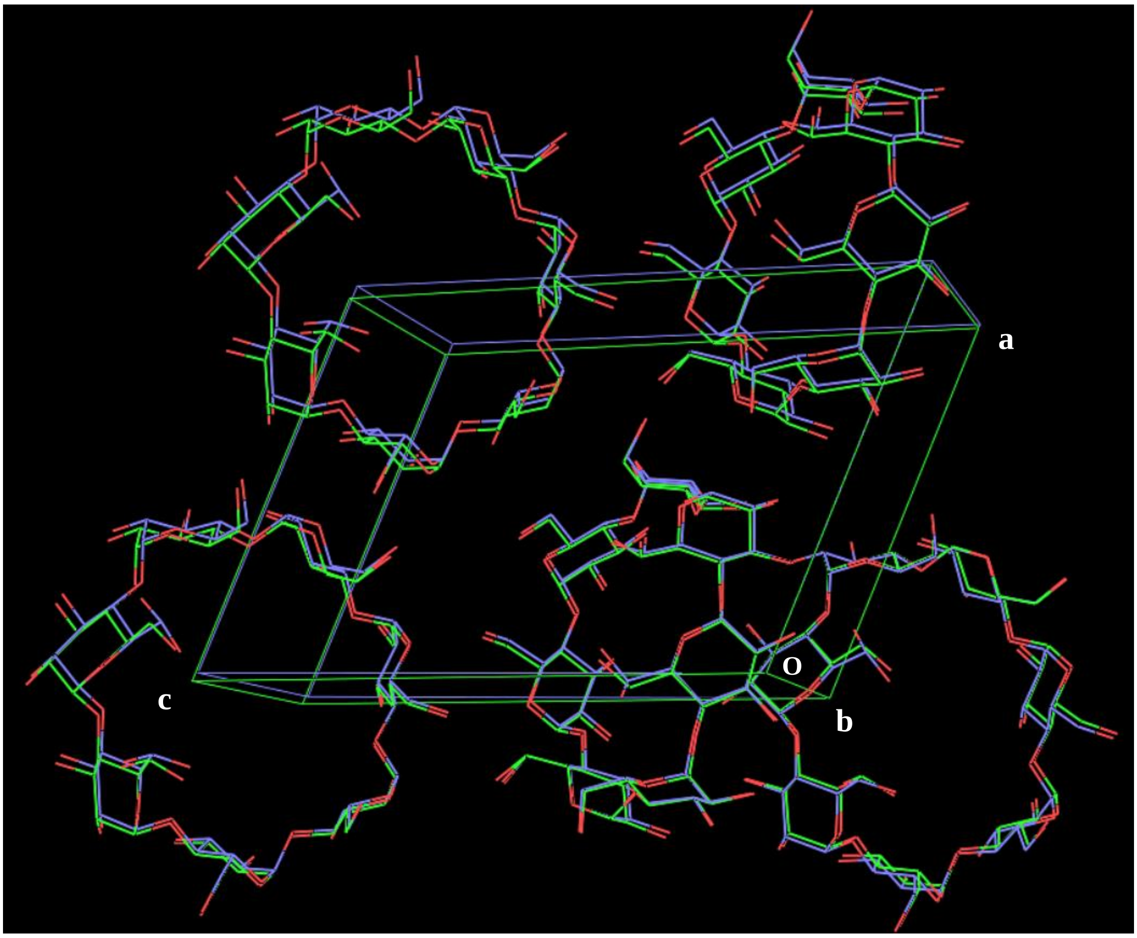

Figure S5: Superposition of β-CD macrocycles in the asymmetric unit of "β-CD–D-NAcTrp" and β-CD–Hydrate clathrate (BUVSEQ01)[2] after (i) transformation of coordinates of BUVSEQ01 **abc** to **c(-b)a** and (ii) change of origin of by 1/2**c** and by 0.429**b** .The two structures do not superposes exactly. The structures were rendered in PyMOL.[5]

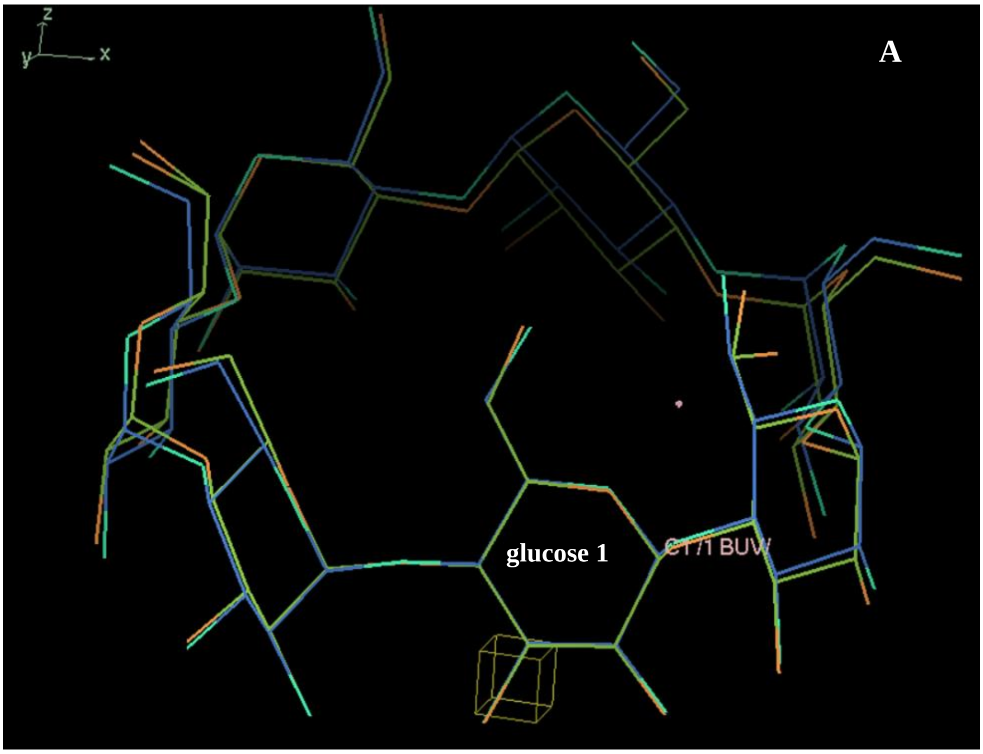
A
glucose 1
C1 /1 BUV

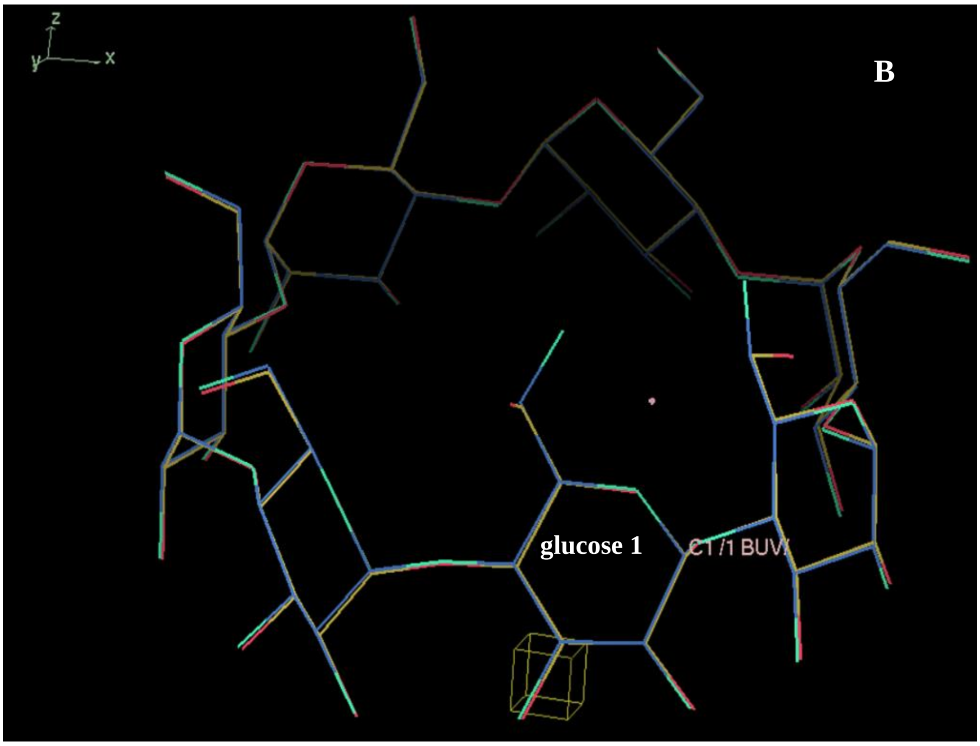
B
glucose 1
C1 /1 BUV

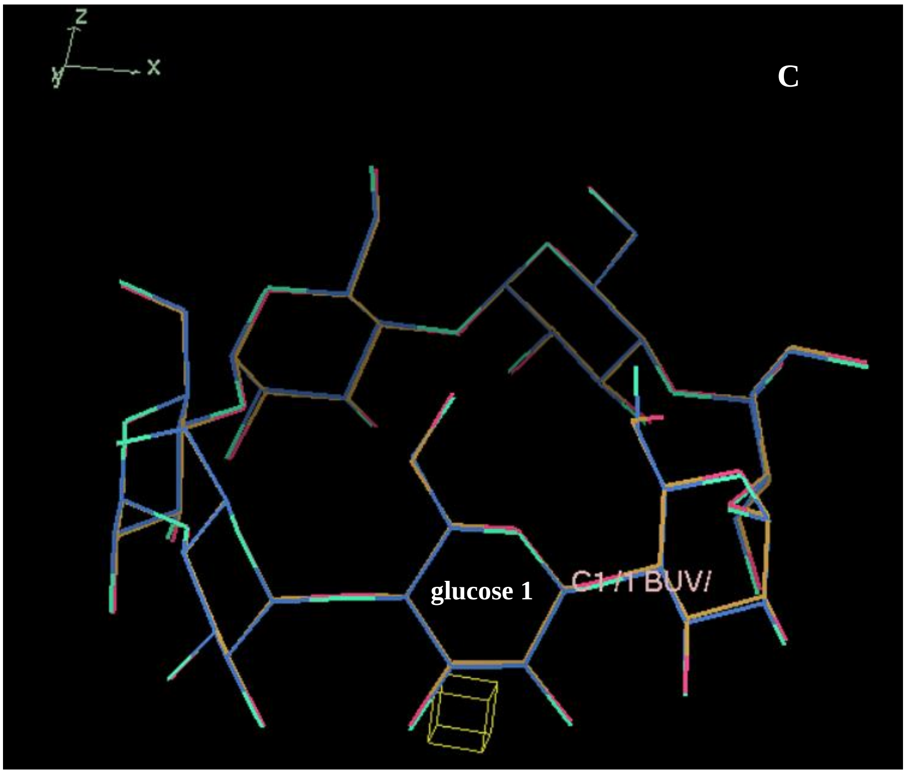

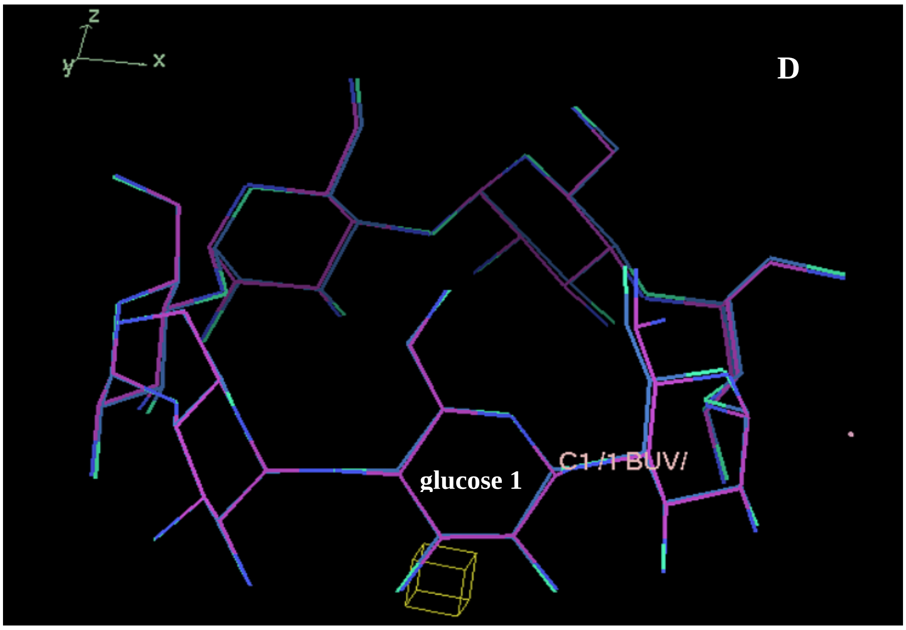

Figure S6: Superposition (rendered in Coot[6]) of one β-CD glucopyranose unit (glucose 1) in pairs of similar structures: (A) present structure "β-CD–D-NAcTrp" (green) and β-CD–Hydrate BUVSEQ01[2] (blue); (B) β-CD–Hydrates BUVSEQ01 (blue) and GAGPOA[4] (yellow); (C) β-CD–Hydrates BUVSEQ01 (blue) and OXAFUQ (orange)[3]; (D) β-CD–Hydrate BUVSEQ01 (blue) and β-CD–glutaric acid[7] (magenta). It is worth noting that in (A) the structures do not superpose exactly, whereas in (B), (C) and (D) they superpose completely.

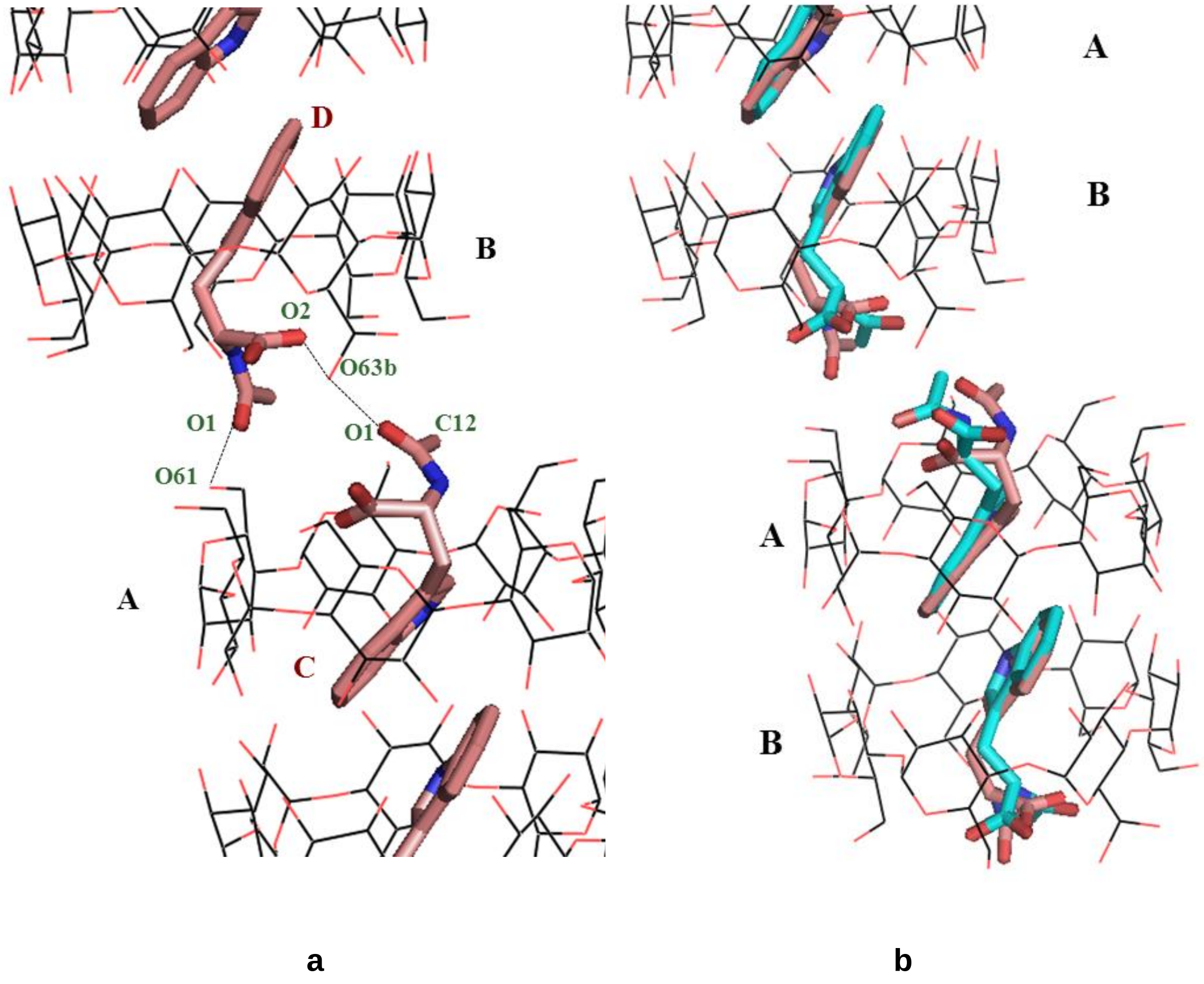

Figure S7: Modeling studies: (a) The D-NAcTrp inside the dimer structure of the β-CD–L-NAcTrp; (b) superposition of the L-NAcTrp (cyan) and D-NAcTrp (pink) inside the β-CD dimer. The structures were rendered in PyMOL.[5]

References

(1) Mercury *Mercury, CSD 1.4.1. New Software for searching the Cambridge Structural Database and visualizing crystal structures*.

(2) Steiner, T.; Koellner, G. *J. Am. Chem. Soc.* **1994**, *116*, 5122.

(3) Ilin, A. *CSD Communication* **2016**, CCDC 1510220.

(4) Ivanova, B.; Spiteller, M. *Int. J. Biol. Macromol.* **2014**, *64* 383.

(5) DeLano, W. L. *The PyMOL Molecular Graphics System, DeLano Scientific LLC, San Carlos, CA, USA*, http://www.pymol.org **2002**.

(6) Emsley, P.; Lohkamp, B.; Scott, W. G.; Cowtan, K. *Acta Crystallog.* **2010**, *D66*, 486.

(7) Paulidou, A.; Yannakopoulou, K.; Mavridis, I. M. *J. Incl. Phenom. Macrocycl. Chem.* **2010**, *68*, 297.
